# Supplementary material for: African savanna elephants (Loxodonta africana) as an example of a herbivore making movement choices based on nutritional needs
Source: PeerJ. 2019 Feb 1;7:e6260. doi: 10.7717/peerj.6260 (PMC6361008; doi:10.7717/peerj.6260)
Supplement: Supplemental Information 1 — Literature reviewed within this review, including peer-reviewed papers and grey literature. [file peerj-07-6260-s001.docx]

**Appendix 1**

**Papers reviewed**

Ben-Shahar R., Coe MJ. 1992. The relationships between soil factors, grass nutrients and the foraging behaviour of wildebeest and zebra. *Oecologia* 90:422–428. DOI: 10.1007/BF00317701.

Birkett PJ., Vanak AT., Muggeo VMR., Ferreira SM., Slotow R. 2012. Animal perception of seasonal thresholds: Changes in elephant movement in relation to rainfall patterns. *PLoS ONE* 7. DOI: 10.1371/journal.pone.0038363.

Bowell RJ., Ansah RK. 1994. Mineral status of soils and forage in the Mole National Park, Ghana and implications for wildlife nutrition. *Environmental Geochemistry and Health* 16:41–58. DOI: 10.1007/BF00209824.

Bowell RJ., Warren A., Redmond I. 1996. Formation of cave salts and utilization by elephants in Mount Elgon region, Kenya. *Environmental Geochemistry and Health* Geological:63–79.

Chiyo PI., Cochrane EP., Naughton L., Basuta GI. 2005. Temporal patterns of crop raiding by elephants: a response to changes in forage quality or crop availability? *Journal of African Ecology* 43:48–55.

Codron J., Lee-Thorp JA., Sponheimer M., Codron D., Grant RC., De Ruiter DJ. 2006. Elephant (*Loxodonta africana*) diets in Kruger National Park, South Africa: spatial and landscape differences. *Journal of Mammalogy* 87:27–34. DOI: 10.1644/05-MAMM-A-017R1.1.

Douglas-Hamilton I., Krink T., Vollrath F. 2005. Movements and corridors of African elephants in relation to protected areas. *Naturwissenschaften* 92:158–163. DOI: 10.1007/s00114-004-0606-9.

Dolmia NM., Calenge C., Maillard D., Planton H. 2007. Preliminary observations of elephant (*Loxodonta africana*, Blumenbach) movements and home range in Zakouma National Park, Chad. *African Journal of Ecology* 45:594–598. DOI: 10.1111/j.1365-2028.2007.00777.x.

de Knegt H., Van Langevelde F., Skidmore A., Delsink A., Slotow R., Henley S., Bucini G., De Boer W., Coughenour M., Grant C., Heitkönig I., Henley M., Knox N., Kohi E., Mwakiwa E., Page B., Peel M., Pretorius Y., Van Wieren S., Prins H. 2011. The spatial scaling of habitat selection by African elephants. *Journal of Animal Ecology* 80:270–281. DOI: 10.1111/j.1365-2656.2010.01764.x.

Hoare R., du Toit J. 1999. Coexistence between people and elephants in African savannas. *Conservation Biology* 13:633–639. DOI: 10.1046/j.1523-1739.1999.98035.x.

Holdø R., Dudley J., McDowell L. 2002. Geophagy in the African elephant in relation to availability of dietary sodium. *Journal of Mammalogy* 83:652–664. DOI: 10.1644/1545-1542.

Holdø RM., McDowell LR. 2004. Termite mounds as nutrient–rich food patches for elephants. *Biotropica* 36:231–239. DOI: 10.1111/j.1744-7429.2004.tb00314.x.

Jachmann H. 1989. Food selection by elephants in the “Miombo” biome, in relation to leaf chemistry. *Biochemical Systematics and Ecology* 17:15–24. DOI: 10.1016/0305-1978(89)90037-9.

Kalumanga E., Mpanduji D., Cousins S. 2017. Geophagic termite mounds as one of the resources for African elephants in Ugalla Game Reserve, Western Tanzania. *African Journal of Ecology*. DOI: 10.1111/aje.12326.

Klaus G., Schmidg B. 1998. Geophagy at natural licks and mammal ecology: A review. *Mammalia* 64:482–498.

Leggett K. 2015. Home range and seasonal movement of elephants in the Kunene Region, northwestern Namibia. *African Zoology* 41:17–36. DOI: 10.1080/15627020.2006.11407332.

Morellet N., Bonenfant C., Börger L., Ossi F., Cagnacci F., Heurich M., Kjellander P., Linnell JDC., Nicoloso S., Sustr P., Urbano F., Mysterud A. 2013. Seasonality, weather and climate affect home range size in roe deer across a wide latitudinal gradient within Europe. *Journal of Animal Ecology* 82:132–139. DOI: 10.1111/1365-2656.12105.

McNaughton S. 1988. Mineral nutrition and spatial concentrations of African ungulates. *Nature* 334:343–345. DOI: 10.1038/334343a0.

McNaughton S. 1990. Mineral nutrition and seasonal movements of African migratory ungulates. *Nature* 345:613–615.

Muvengwi J., Mbiba M., Nyenda T. 2013. Termite mounds may not be foraging hotspots for mega-herbivores in a nutrient-rich matrix. *Journal of Tropical Ecology* 29:551–558. DOI: 10.1017/S0266467413000564.

Muvengwi J., Ndagurwa H., Nyenda T., Mlambo I. 2014. Termitaria as preferred browsing patches for black rhinoceros (*Diceros bicornis*) in Chipinge Safari Area, Zimbabwe. *Journal of Tropical Ecology* 30:51–598. DOI: 10.1017/S0266467414000480.

Mwangi P., Milewski A., Wahungu G. 2004. Chemical compsition of mineral licks used by elephants in Aberdaes National Park, Kenya. *Pachyderm* 37:59–67.

Osborn F V. 2004. Seasonal variation of feeding patterns and food selection by crop-raiding elephants in Zimbabwe. *african Journal of Ecology*:322–327.

Pretorius Y., de Boer F., van der Waal C., de Knegt H., Grant R., Knox N., Kohi E., Mwakiwa E., Page B., Peel MJ., Skidmore A., Slotow R., van Wieren S., Prins H. 2011. Soil nutrient status determines how elephant utilize trees and shape environments. *Journal of Animal Ecology* 80:875–883. DOI: 10.1111/j.1365-2656.2011.01819.x.

Pretorius Y., Stigter JD., de Boer WF., van Wieren SE., de Jong CB., de Knegt HJ., Grant CC., Heitkönig I., Knox N., Kohi E., Mwakiwa E., Peel MJS., Skidmore AK., Slotow R., van der Waal C., van Langevelde F., Prins HHT. 2012. Diet selection of African elephant over time shows changing optimization currency. *Oikos* 121:2110–2120.

Rode K., Chiyo P., Chapman C., McDowell L. 2006. Nutritional ecology of elephants in Kibale National Park, Uganda, and its relationship with crop-raiding behaviour. *Journal of Tropical Ecology* 22:441. DOI: 10.1017/S0266467406003233.

Shannon G., Page B., Mackay R., Duffy K., Slotow R. 2008. Activity Budgets and Sexual Segregation in African Elephants (*Loxodonta africana*). *Journal of Mammalogy* 89:467–476. DOI: 10.1644/07-MAMM-A-132R.1.

Sienne JM., Buchwald R., Wittemyer G. 2014. Differentiation in mineral constituents in elephant selected versus unselected water and soil resources at Central African bais (forest clearings). *European Journal of Wildlife Research* 60:377–382. DOI: 10.1007/s10344-013-0781-0.

Teitelbaum C., Fagan W., Fleming C., Dressler G., Calabrese JM., Leimgruber P., Mueller T. 2015. How far to go?: Determinants of migration distance in land mammals. *Ecology Letters* 18:545–552. DOI: 10.1111/ele.12435.

Thomas B., Holland J., Minot E. 2008. Elephant (*Loxodonta africana*) home ranges in Sabi Sand reserve and Kruger National Park: A Five-year satellite tracking study. *PLoS ONE* 3. DOI: 10.1371/journal.pone.0003902.

Tucker MA., Böhning-Gaese K., Fagan WF., Fryxell JM., Van Moorter B., Alberts SC., Ali AH., Allen AM., Attias N., Avgar T., Bartlam-Brooks H., Bayarbaatar B., Belant JL., Bertassoni A., Beyer D., Bidner L., van Beest FM., Blake S., Blaum N., Bracis C., Calabrese JM., Ford AT., Fritz SA., Gehr B., Goheen JR., Hof C., Hurme E., Kaczensky P., Kane A., Kappeler PM., Kauffman M., Leimgruber P., C Linnell JD., López-López P., Catherine Markham A., Morato RG., Morellet N., Morrison TA., Nandintsetseg D., Nathan R., Niamir A., Odden J., O RB., Gustavo Oliveira-Santos LR., Olson KA., Patterson BD., Rimmler M., Rogers TL., Moe Rolandsen C., Rosenberry CS., Zięba F., Zwijacz-Kozica T., Mueller T. 2018. Moving in the Anthropocene: Global reductions in terrestrial mammalian movements. *Science* 359:466–469. DOI: 10.1126/science.aam9712.

Weir JS. 1969. Chemical properties and occurrence on Kalahari sand of salt licks created by elephants. *Journal of Zoology* 158:293–310.

Weir JS. 1972. Spatial Distribution of Elephants in an African National Park in Relation to Environmental Sodium. *Oikos* 23:1–13.

Western D., Lindsay W. 1984. Seasonal herd dynamics of a savanna elephant population. *African Journal of Ecology* 22:229–244. DOI: 10.1111/j.1365-2028.1984.tb00699.x.

Whitehouse A., Schoeman D. 2003. Ranging behaviour of elephants within a small, fenced area in Addo Elephant National Park, South Africa. *African Zoology* 38:95–108. DOI: 10.1080/15627020.2003.11657197.

**Grey literature**

Begon M., Harper J., Townsend C. 1986. *Ecology: Individuals, populations and communities*.

Blake S. 2002. The ecology of forest elephant distribution and its implications for conservation. University of Edinburgh.

Dierenfeld E. 2008. Biology, medicine, and surgery of elephants. In: Fowler M, Mikota SK eds. *Biology, medicine, and surgery of elephants*. Wiley-Blackwell publishing,. DOI: 10.1002/9780470344484.

Ellis R. 1990. *Tannin-like substances in grass leaves*. Pretoria: National Botanical Institute, South Africa.

Nelson L. 1981. *Secondry hypocuprosis in an exotic animal park*. San Diego.

Partington C. 2012. Feeding, nutrition and body condition of UK elephants. University of Liverpool.

Sukumar R. 1990. Ecology of the Asian elephant in southern India. II. Feeding habits and crop raiding Patterns. *Journal of Tropical Ecology* 6:33–53. DOI: 10.2307/2559367.

Underwood E. 1977. *Trace elements in human and animal nutrition*. Academic Press.
